# Silver Nanoparticles Improve Fluorophore Photostability: Application to a Hypericin Study

**DOI:** 10.3390/ijms25189963

**Published:** 2024-09-15

**Authors:** Grzegorz Wołąkiewicz, Monika Pietrzak, Mariusz Szabelski

**Affiliations:** Department of Physics and Biophysics, University of Warmia and Mazury, 10-719 Olsztyn, Poland

**Keywords:** silver nanoparticles, fluorescence, photobleaching, photoreaction, hypericin

## Abstract

Protection against the negative effects of solar radiation involves using cosmetics with a UV filter, but visible radiation can also have negative effects. We use dietary supplements and take medications; unfortunately, many of them contain substances that degrade under the influence of visible light, which transform into chemical compounds harmful to health. Manufacturers often include information on the prohibition of exposure to sunlight on the packaging, but consumers often do not read the product leaflet. The solution to this problem may be the addition of silver particles to preparations. In the presented article, we proposed the use of silver nanoparticles to reduce the photobleaching and photoreaction of fluorophore, while increasing the fluorescence intensity. For our research, we used a compound that is particularly sensitive to radiation: hypericin.

## 1. Introduction

Exposure to sunlight can be dangerous not only because of UV rays. Every day, we take various medications and dietary supplements which often contain various photosensitizing substances that react to the whole spectrum of light. Therefore, it is important to work on increasing the photostability of photosensitizers, which will prevent the formation of unfavorable compounds resulting from their transformation under the influence of light. One of such compounds with many health-promoting properties is hypericin, which undergoes photodegradation when exposed to light. Hypericin is a plant-derived substance with many different medicinal properties. It has been used in herbal medicine, and also has antiviral, antibacterial, anticancer [1,2] and antidepressant properties [3,4]. Due to its structure—a large system of flat rings—it can be classified as an interceptor molecule [5]. Intercepting leads to a decrease in the concentration of an active form of mutagens and prevents their negative effects on the body [6,7]. Many shops and pharmacies sell preparations containing hypericin in the form of dried herbal St. John’s wort (Hypericum perforatum) for preparing teas and infusions, as well as dietary supplements containing St. John’s wort extract as a hard tablets, gel capsules or drops. Unfortunately, hypericin is not soluble in water, which is its disadvantage. Therefore, many works on hypericin focus on improving its solubility in water, which facilitates its therapeutic use [8,9]. Additionally, hypericin is also a very strong natural photosensitizer. After light excitation, electron transfer takes place in both the singlet [10] and triplet [11] states. Mechanisms of all processes taking place in the light are not fully understood. As a result of these processes, singlet oxygen and superoxide anion are formed with high efficiency. This feature allows for hypericin to be used as a photosensitizer in photodynamic therapy applied for cancer treatment (PDT) [12]. The double nature of hypericin is noted by Jendželovská at al. (2016), in the work entitled “Hypericin in the Light and in the Dark: Two Sides of the Same Coin” [13]. Hypericin shows different properties in the dark and different in the light. Therefore, when we want to use hypericin in a role other than a photosensitizer, for example, as a mild antidepressant or an interceptor molecule, photolability is definitely undesirable.

Hypericin emits red fluorescence which allows for the process in the excited state to be monitored [14]. Improvement in the photostability of hypericin can be achieved by the use of noble metal nanoparticles (NPs). Nanoparticles of silver and gold exhibit strong absorption bands that are absent in the bulk metals. The enhanced fluorescence on the surface of metals is due to the following: local field enhancement near the metal surface-localized surface plasmon resonance (LSPR), plasmon coupling and the effect of radiative decay engineering (RDE) [15]. RDE is related to fluorescence amplification by changing the rate of radiative and non-radiative decay, leading to emission amplification [16]. Localized surface plasmon resonance is associated with the interaction of light of a specific wavelength with oscillating electrons on the surface of metal nanoparticles. As a result of the interaction, we observe a collective oscillation of electrons and, as a result of the resonance phenomenon, the creation of a local electromagnetic field on the metal surface, leading to higher-emission intensity and changes in the optical properties of the fluorophore. A generated electric field around the metal can interact with the fluorophore and affect its emission, causing a bidirectional interaction. The consequence of these phenomena may be increased photostability due to shorter lifetimes, increased quantum efficiency, and a reduced signal background level [17]. The enhancement will be observed for a very close distance between the fluorophore and the surface of the metal nanoparticles (<90 nm) and a specific size of nanoparticles with diameters much smaller than the excitation light wavelength [18].

Due to the large surface-to-volume ratio of noble metal nanoparticles compared to atoms or molecules of the same material, they have unique optical, chemical and mechanical properties for medical applications [19,20]. The nanoparticles of silver, gold, zinc, copper and titanium have unique antimicrobial properties. Nanoparticles of these metals can undergo a reaction leading to the formation of free radicals (reactive nitrogen species, RNS) and trigger cytotoxicity in bacterial cells. Studies indicate the anticancer potential of silver (AgNP) and gold (AuNP) nanoparticles as a result of the formation of RNS inside cells. The selectivity of the nanoparticles was demonstrated by releasing the death-determining silver ions only into carcinogenic cells [21]. The most commonly used nanoparticles are AgNP and AuNP, but silver nanoparticles are considered to be more useful for applications and producing better results [22]. Due to their high biocompatibility and chemical stability, they are used to design modern biosensors and in research on new methods of delivering drugs to the body or phototherapy [23].

In the presented work here, we attempted to stabilize hypericin exposed to light. We used the phenomenon of fluorescence enhancement on the surface of metals. The possibilities of detecting fluorophores are often limited due to their different photostability, quantum yields or autofluorescent properties of the tested samples [24]. For this reason, enhanced fluorescence techniques on the surface of metal nanoparticles (metal-enhanced fluorescence, MEF) can be used to increase sensitivity and allow to detect even very low concentrations of hypericin in the samples. Additionally, thanks to the presence of silver, preparations containing hypericin will be protected against bacterial growth and will remain in the excited state for a shorter period of time reduces the time for oxidative processes, which directly increases the photostability.

## 2. Results and Discussion

**Figure 1:**
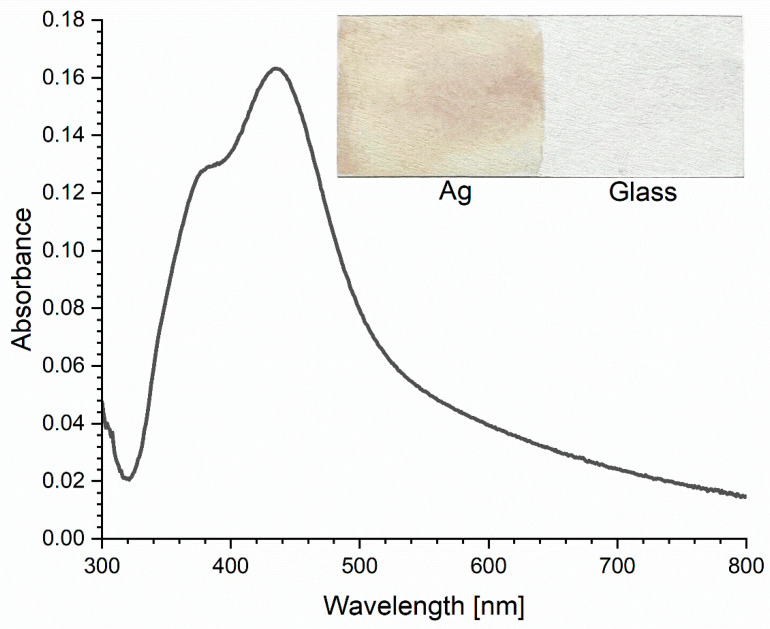
Absorption spectrum of a slide with silver nanoparticles.

Silver nanoparticles have the ability to absorb and scatter light, which makes it possible to control the size of the synthesized particles by determining the location of the absorption band maximum in the range of 400–530 nm. To obtain fluorescence enhancement, a specific spectral shape and position are expected. In this study, we chose to use a silver colloid nanolayer to avoid strong scattering observed in colloidal suspensions. The absorption spectrum of our nanosilver layer (Figure 1) is consistent with the literature data [25,26].

**Figure 2:**
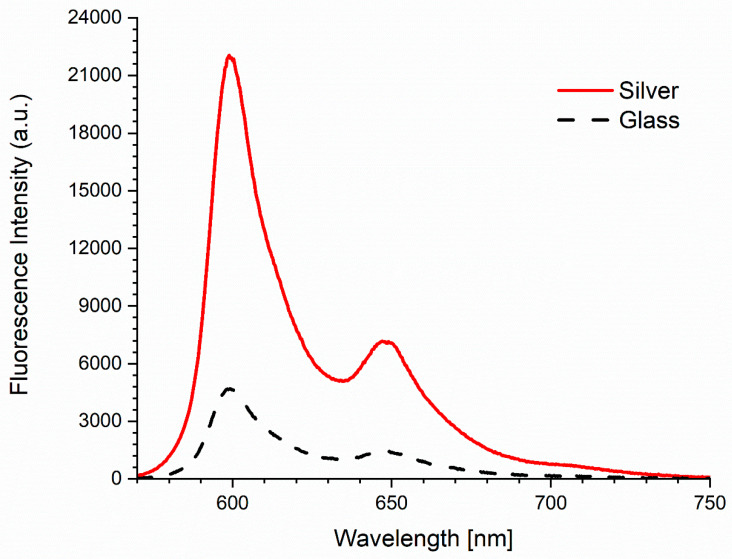
Fluorescence enhancement of hypericin on the layer of silver nanoparticles.

The enhancement of the hypericin fluorescence emission on the nanosilver layer in relation to the signal on the glass was observed. The signal on the nanosilver layer for a 600 nm wavelength was 4.7 times higher than on the glass and from the calculation of AUC (for spectrum between 570 and 750 nm)—4.8 times (Figure 2).

**Figure 3:**
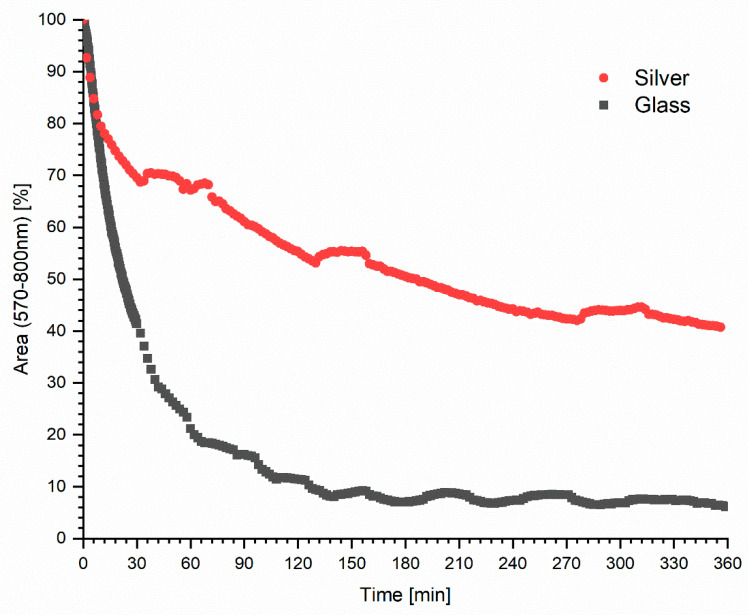
Fluorescence intensity of hypericin in time on the glass and a layer of silver nanoparticles as a function of the fluorescence spectrum area in the 570–800 nm range.

As demonstrated in the study by Matveeva et al., the level of fluorescence enhancement for different fluorophores associated with various objects on the surface of silver nanoparticles may vary from several to several dozen times in relation to the signal on the glass itself [27]. The silver colloids amplify the fluorescence signal of fluorophores of which the excitation and emission is in the visible light range [24,27,28,29]. The presence of silver nanoparticles affects the stability of the fluorophore emission (Figure 3). In order to present the results, the ratio of the area under the curve (570–800 nm) at exposure time *t* to the area at the initial time *t_0_* was calculated in percentage terms. The initial value of the fluorescence intensity decreased by 90% in the first two hours of exposure for laser light hypericin on the glass-only layer. However, on the nanosilver layer, after 2 h of exposure, the decrease in fluorescence intensity of fluorophore was only 45%, and at the end of the experiment, after 6 h, the fluorescence signal was still strong at 40% of the initial value. The significant difference in the value and dynamics of the decrease in intensity between exposure on the glass and nanosilver layer confirms better stability of this compound on silver. The presence of hypericin in a highly concentrated electrical field with a localized charge density oscillations created by AgNP causes a simultaneous shortening of the lifetimes in the excited state that increase photostability. The decreased lifetimes with increased intensities suggest an increased radiative rate for the affected hypericin, allowing it undergo more excitation–deexcitation cycles prior to photodecomposition. As the time-zero intensities are much higher with the AgNP, the number of observable photons per fluorophore increased dramatically, because the rate of photobleaching is lower in the presence of silver nanoparticles. Furthermore, the presence of hypericin chromophores in the excited state for a shorter time reduces the time for oxidative processes, which directly increases the photostability and prevents photodegradation processes and the formation of products potentially harmful to human health.

**Figure 4:**
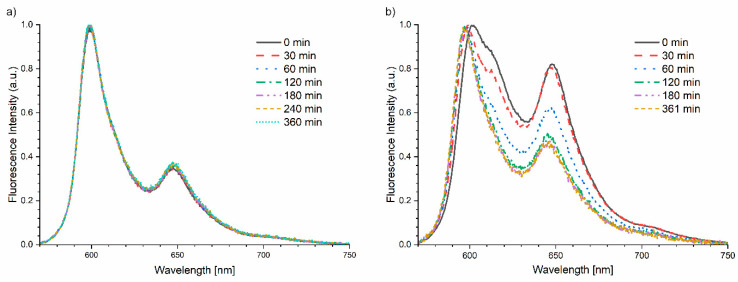
Normalized fluorescence signal of hypericin on silver (**a**) and glass (**b**) at different exposure times.

**Figure 5:**
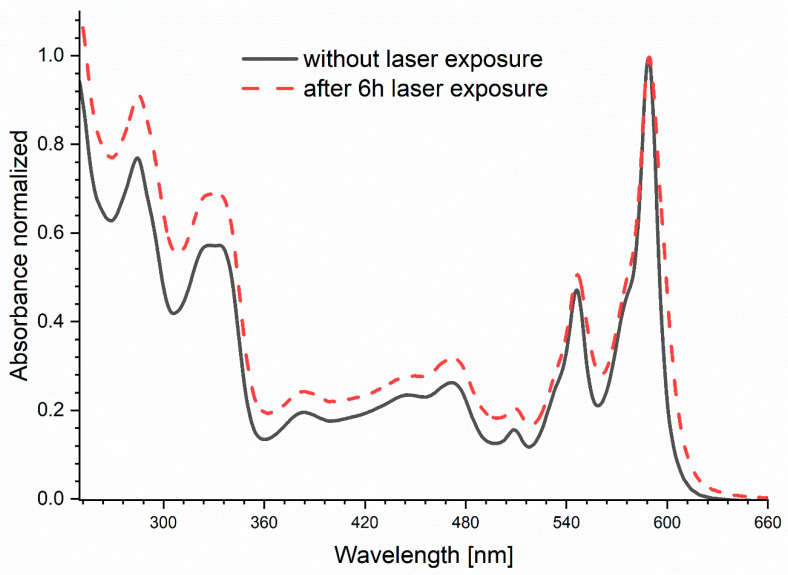
Normalized absorption spectrum of hypericin before and after irradiation on glass.

As shown in Figure 4a, there are no changes in the shape, position and the ratio between 600 and 650 nm bands for hypericin on the silver layer. We observed only signal decreasing, caused by the photobleaching effect. Additional photoreaction effects can be observed for hypericin on the glass (Figure 4b), as indicated by large changes in relations between the 600 and 650 nm bands. After long-term exposure for the light of hypericin on glass, the ratio of the normalized fluorescence intensity for the 600 nm band (1st peak) to the intensity for the 650 nm band (2nd peak) F1/F2 was almost doubled (Table 1). We also observed a spectral shift toward lower wavelengths up to 6 nm. The photoreaction effects shown above were not visible in case of hypericin on the nanosilver layer. The relationship between the 600/650 nm bands remained constant during irradiation, and the maxima of fluorescence intensity were recorded for the same wavelengths. Additionally, it should be noted that in the case of irradiation of the hypericin solution on the glass, the emission spectrum collected at time *t_0_* has a different shape and position compared to the spectra recorded on the nanosilver layer. During exposure, the spectrum changes to finally obtain a shape and position identical to the spectra recorded on the silver surface. The obtained results indicate that on silver in the excited state, there is immediate stabilization and a shift of the equilibrium toward one stable form of hypericin, while in the solution placed on glass we are dealing with various forms of hypericin which undergo photoreaction during exposure. The stable form of hypericin undergoes only the photobleaching process. This photoreaction effect is also visible on the absorption spectrum (Figure 5) as the ratios of the individual absorption bands changed, before and after exposure to the light.

There was a pronounced change in the calculated lifetimes for main component τ_1_ (Table 2). For both observations at 600 and 650 nm, before exposure, τ_1_ values were the same. After laser exposure, long-lived lifetime components were shortened by 100 ps, with a slight change in percentages of these components (α_1_), also confirming the photoreaction effects of hypericin on a glass surface. 

The processes occurring under the influence of light in the hypericin molecule are still not fully explained. It is known that more than one process can take place here [30,31]. The basic phenomenon is the excitation of the hypericin molecule to the first singlet state and then transition to the triplet state. From the triplet state, energy transfer to ground-state oxygen and production of singlet oxygen can occur [32]. This process is associated with the ~5 ns component of the hypericin fluorescence spectrum and is responsible for the photobleaching effect [33]. The nanosilver layer definitely slows down photobleaching. Since no changes in the shape of the fluorescence spectra were observed, most likely no other processes are taking place here. Additionally, shorter lifetimes were recorded and are related to processes involving changes in the chemical structure of hypericin that can occur in the excited state, e.g., intramolecular transfer of the hydrogen atom and formation of tautomers. The appearance of the signal from short-lived individuals and their values strongly depend on the power and frequency of the laser and the medium in which hypericin is located [1,2,30,31,33,34]. Plaza et al. [31] note that structural changes in the molecule only appear at higher excitation energies and suggest that this can also be explained by partial multiphotonic excitation. Changes in the fluorescence and absorption spectra of hypericin solution exposed to laser light on the glass suggest complicated processes in the excited state, leading to photodegradation.

## 3. Materials and Methods

The tested matrix consisted of cover slips purified and covered by applying approx. 1 mL of a Poly-L-lysine solution with phosphate buffer (0.01 M). Silver nanoparticles were chemically synthesized, to form silver islands on the surface of the slides [25]. In the synthesis, we used as a metal precursor, silver salt (AgNO_3_, ≥99%, Sigma-Aldrich, St. Louis, MO, USA) and reducing agent–D (+) glucose (≥99.5% GC, Sigma-Aldrich, St. Louis, MO, USA). To intensive stirring solution of 60 mL deionized water and 500 mg AgNO_3_, 750 μL–5% NaOH were added. After obtaining a solution with a brown precipitate, added slowly, drop by drop, approx. 2 mL–30% NH_4_OH, until the solution was clear. The mixture was cooled down to a temperature of about 2 °C in an ice bath. Then, 720 mg of D (+) glucose, dissolved in 15 mL of deionized and chilled water, was added to the reaction solution and mixed for 2 min. Four slides, in two pairs, were inserted into the solution and heated to approx. 24 °C for 3 min, until the color of the slides become yellow-greenish. Slides were removed and rinsed with clean water. The silver colloid islands formed on the glass surface were monitored by measuring the absorption spectra with a Varian Cary 50 scan.

The stock solution of hypericin was obtained by dissolving a portion of hypericin (0.38 mg) in 1.5 mL DMSO (Dimethyl sulfoxide, ≥99.9%, Sigma-Aldrich, St. Louis, MO, USA). The actual test solution was prepared by diluting a 60 µL stock solution with 1.44 mL of glycerol (Sigma-Aldrich, St. Louis, MO, USA). To evaluate the enhancement of the fluorescence of hypericin on the surface of silver nanoparticles, a drop of test solution was applied to a glass slide with and without a nanosilver layer and covered with a second coverslip, creating a very thin film of fluorophore solution on the test surface. The samples were exposed to a 530 nm laser LDH-D-TA-530 PicoQuant GmbH, Berlin, Germany working in continuous mode, with maximum power of 10 mW. Spectra were recorded for 6 h with an FLAME-S-UV/NIR-ES Ocean Optic, Orlando, FL, USA miniature spectrometer, equipped with a filter that cuts off the excitation laser signal.

The area under curve (AUC) was calculated in Origin 2022 b software. Additionally, the lifetimes of hypericin solution before and after exposure to laser light were measured using the time-domain spectrometer FluoroTime 200, PicoQuant GmbH, Berlin, Germany equipped with an R3809 U-50 micro-channel plate photomultiplier (MCP-PMT, Hamamatsu, Hamamatsu City, Japan), PicoHarp300 TCSPC module and PDL800-D driver PicoQuant GmbH, Berlin, Germany. Fluorescence was excited with a 530 nm using the same PicoQuant laser working in pulsed mode, and observed at 600 and 650 nm.

## 4. Conclusions

The presence of silver nanoparticles causes a shift in the equilibrium between the various forms of hypericin present in the solution toward a stable form that does not undergo photoreaction but only photobleaching in the excited state. Moreover, silver causes an approximately five-fold increase in the fluorescence intensity of hypericin and the photobleaching process is much slower. The use of nanosilver for research and applications involving hypericin will allow for better detection and work with the dominant stable form of hypericin, which is of great importance because currently, we cannot clearly state in what forms it occurs in aqueous solution, what reactions hypericin undergoes and what are the products of these reactions.

The obtained results can be used by drug manufacturers to develop new and much safer-to-use medical preparations. Increasing the stability of hypericin will extend the health-promoting effect, and the addition of silver will prolong the durability of the preparations thanks to the antibacterial properties of Ag.

## Figures and Tables

**Table 1:** Change in the relationship between the position of normalized spectra and fluorescence intensity for bands characteristic for hypericin 600 and 650 nm after exposure to light on glass and a nanosilver layer.

Exposure Time [min]			0	30	60	120	180	361
**Glass**	1st peak	Position [nm]	602.3	599.3	597.6	596.9	596.6	596.3
		F1 Intensity [a.u.]	1	1	1	1	1	1
	2nd peak	Position [nm]	649.0	646.4	647.4	645.1	646.4	647.4
		F2 Intensity [a.u.]	0.82	0.81	0.63	0.51	0.47	0.48
	**F1/F2**		**1.22**	**1.24**	**1.59**	**1.96**	**2.12**	**2.08**
**Silver**	1st peak	Position [nm]	598.6	598.3	598.6	598.3	598.6	598.9
		F1 Intensity [a.u.]	1	1	1	1	1	1
	2nd peak	Position [nm]	647.4	647.4	647.0	648.3	648.7	647.4
		F2 Intensity [a.u.]	0.35	0.36	0.36	0.36	0.36	0.38
	**F1/F2**		**2.86**	**2.79**	**2.76**	**2.76**	**2.76**	**2.64**

**Table 2:** Calculated fluorescence lifetime of hypericin before and after 6 h exposure to laser light on glass. τ_i_—fluorescence lifetime; α_i_—pre-exponential factor; τ_av_—amplitude weighted average lifetime; χ^2^—goodness of fit.

Hypericin	Observation [nm]	τ_1_ [ns]	α_1_ [%]	τ_2_ [ns]	α_2_ [%]	τ_av_ [ns]	χ^2^
Not exposed	600	5.153 ± 0.018	81.91	2.576 ± 0.107	18.09	4.687	1.027
6 h of exposure		5.058 ± 0.017	87.73	2.702 ± 0.159	12.27	4.769	1.009
Not exposed	650	5.162 ± 0.017	91.74	2.925 ± 0.243	8.26	4.978	1.017
6 h of exposure		5.070 ± 0.017	93.25	2.970 ± 0.291	6.75	4.929	1.017

## Data Availability

Data are contained within this article.
